# LIMITED JOINT MOBILITY IN A CHILD WITH TYPE 1 DIABETES MELLITUS.

**DOI:** 10.1155/2021/6397338

**Published:** 2021-11-15

**Authors:** Mohammad H. Al-Qahtani, Fai A. AlQahtani

**Affiliations:** Department of Pediatrics, Collage of Medicine, King Fahad Hospital of the University, Imam Abdulrahman Bin Faisal University, Alkhobar, Saudi Arabia

## Abstract

Chronic uncontrolled type 1 diabetes mellitus (type 1DM) is a very major risk for chronic systemic complications; specifically, the microvascular and macrovascular ones. Limited joint mobility (LJM) is a rare disease that complicates all types of diabetes and might indicate the high-risk odd for the diabetic patients to develop microvascular complications. We are reporting a 13-year-old female child with chronic uncontrolled type 1DM presenting with full blown clinical picture of bilateral hand LJM associated with significant growth failure yet has no clinical or biochemical evidence of microvascular complications. Literature research studies have emphasized the rarity of this manifestation in pediatric type 1 diabetic patients; however, it is an important clue and warning sign for microvascular complication occurrence in these patients.

## 1. Introduction

Limited joint mobility (LJM) previously known as cheiroarthropathy or stiff hand syndrome is a progressive flexion deformity which affects mainly hand digits and sometimes associated with thickened waxy and tight overlying skin and characterized clinically by painless limited extension of the proximal metacarpophalangeal joints and/or interphalangeal joints with fixed flexion of the fingers [1]. It is the most frequent joint-related disease in adult diabetic patients. Although it is seen in nondiabetic patients, multiple digit involvement is more prevalent in diabetic patients compared with the nondiabetic control group [2].

In diabetes mellitus, its pathology is due to progressive nonenzymatic glycation of the collagen making it resistant to the collagenase enzyme leading to capsular thickening and dermal fibrosis with end result of loss of the connective tissue elasticity and might be complicated by joint deformity, found to be correlated with the duration of poor glycemic control and microvascular complications. [3].

It was initially thought to be seen in juvenile type 1 diabetic patients with growth failure; however, it is considered an early complication in both types of diabetes and affects all the ages [1]. It is prevalent in adult diabetic patients with peripheral neuropathy or diabetic foot complications. In one study, all diabetic patients with LJM were found to have clinical evidence of microvascular complications [4]; that is why, it is important to diagnose it as it might be the earlier warning signal for the presence of one or more of the microvascular complications such as nephropathy, retinopathy, and neuropathy.

Generally, this disorder might improve by strict glycemic control and physiotherapy.

We are reporting an adolescent female with long standing uncontrolled type 1 diabetes mellitus presenting with the typical history and clinical signs of bilateral hand limited joint mobility disease.

## 2. Case Report

A 14-year-old female with type 1 diabetes mellitus (type 1 DM) since the age of 6 months, reported progressive painless stiffness in both hands over two-year duration, progressed into limited movements of all hands' joints including the wrist and interphalangeal joints associated with tightening of the overlying skin but without evidence of Raynaud phenomenon.

Her diabetes had been always poorly controlled with glycosylated hemoglobin (HbA1c) ranging between 10 and 12%. Although she is on multiple daily injection insulin regimen with appropriate total daily dose, she has poor compliance to her medication in addition to poor diet control with frequent hospital admissions due to diabetic ketoacidosis (DKA). Two years earlier, she had been investigated for delayed-bone-age short stature including growth hormone stimulation test which became sufficient. Despite her poor glycemic control, she had no evidence of nephropathy or retinopathy. The patient had no symptoms of hypothyroidism, adrenal insufficiency, or polyglandular autoimmune disease; furthermore, no history suggestive of Celiac disease and its yearly screening test is within normal limit. The patient had no history of multiple joint pain, swelling, or hotness; no history of limping or morning stiffness; and no history of intermittent fever or weight loss. Family history is significant for type 2 diabetes mellitus in both parents. However, no family history of microvascular complications and no arthropathy disease or rheumatological disorders.

**Figure 1:**
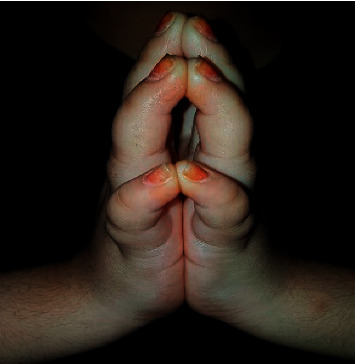
“Prayer” sign showing the significant involvement of the JML of both hands limiting the palmar digits' alignment on each other. The shiny tight skin over the digits is also shown.

Clinical examination showed 3 SDS short-stature pubertal female with stable vital signs. Hand inspection showed flexure contracture of both hands with thick, tight, waxy skin over the dorsal aspect of metacarpophalangeal joints (MCPJ) and interphalangeal joints (IPJ) with normal nail structure and color. Clinically, there was nontender swelling over both hands with no hotness or dislocated joints. Limited joint mobility was confirmed clinically with the “Prayer sign” (Figure 1) (written consent was signed by parents), manifested as incomplete approximation of all of the palmar surfaces of her both hands' digits. She also had positive “table top” sign, not able to completely lay her palms flat on the horizontal surface of the flat table, with no clinical signs of peripheral neuropathy or peripheral vascular disease of the feet. The patient was clinically euthyroid with no thyromegaly and had no clinical signs of adrenal insufficiency or hypoparathyroidism excluding autoimmune polyglandular syndrome type 1(APS-1). No clinical stigmata of systemic lupus or juvenile rheumatoid arthritis.

Retinal examination done by an ophthalmologist did not reveal any retinopathic changes.

Laboratory tests showed a high glycosylated hemoglobin c (HbA1c) level of 12.3%, normal thyroid function tests, and normal microalbumin test with urine albumin/creatinine of 20 mg/g. The patient had normal CBC with normal inflammatory markers.

As for management, the insulin total dose was increased by 15%, and the patient was offered a continuous glucose monitoring device to improve her glycemic control with strict follow-up visits to diabetic nutrition clinic to better manage her dietary intake. She was referred to pediatric orthopedic service for management of her hands' limitation of movement and started on physiotherapy and occupation therapy to improve her hands' range of motion and to increase her muscle strength and functional performance.

## 3. Discussion

Our patient is having type 1 DM for more than 13 years, which is well known for its acute and chronic biochemical, pathophysiological, and functional complications; one of them is limited joint mobility LJM [5]. Although the differential diagnoses of joint involvement in female children are wide, most of the rheumatological, orthopedic, and systemic diseases were excluded in our patient based on the history, physical examination, and relevant laboratory tests. LJM has declined in frequency over two decades from 1970 till 1990 which was attributed to the better glycemic control offered by the availability of effective insulin therapy regimen [6]. This child developed this complication after a long duration of diabetes. Diabetic children might have LJM as early as few months of onset of type 1 DM [7], compared to the late peak prevalence of LJM in 65% of DM generally after 30 years of onset [2]. LJM was considered as a measure of the diabetes control quality [6]. Our patient was having a high level of HbA1c as well as day to day home glucose readings, despite her adjusted insulin dose, indicating its correlation with chronic poor diabetes. Compared to our patient, we reported LJM in a 14-year-old male with chronically uncontrolled diabetes associated with celiac disease, receiving old mixed insulin regimen, yet he did not have evidence of retinopathy nor nephropathy [8].

Despite the chronic poor control disease, our patient developed neither retinopathy nor nephropathy. Although literature is not conclusive in the LJM value as preceding the retinopathy and nephropathy, it is recommended to be part of clinical assessment of type 1DM patients as it might worn the presence of microvascular complications sooner. In different studies, they found significant association of LJM and later development of retinopathy and nephropathy in diabetic patients regardless of the duration of the disease [5, 6].

Limitation of hand functionality in our patient manifested as restricted flexion and extension of the hand fingers which is explained by the connective tissue disturbance affecting the palmar aspects of the hands as mentioned in the literature. Our patient had positive findings in the two clinical tests of LJM; “Tabletop” sign with moderate severity and positive “Prayer” sign both tests are considered helpful tools in the clinical assessment. However, the prayer sign is more sensitive compared to the “Tabletop” because it detects the early pathological changes [9].

The growth failure is one of the features of poorly chronic diabetic children and adolescents. Our patient was short with poor growth velocity and had normal peak growth hormone level. However, poor glycemic control is affecting the growth hormone response at the tissue level. Oxford study found an association of LJM with growth impairment in chronic poorly controlled diabetic children [7].

Our patient had long stay type 1 DM with persistent high HbA1c level, which was found to be significantly correlated with high serum concentrations of advanced glycation endproducts (AGE) which is responsible for skin collagen glycation and consistent with the early appearance of LJM and its correlation with long-term control.

Although LJMS is considered as an irreversible disorder and has no curative therapeutic options, the main stay of treatment is symptomatic medications ranging from simple, nonsteroidal anti-inflammatory drugs to local corticosteroid injections to relieve the pain and to decrease tendinitis or ameliorate the flexor tendon contractures. Surgical intervention is limited to severe contractures.

Physiotherapy intervention targeting stretching exercises of the palm of the hand and fingers will help to prevent progression of the joint stiffness in these cases [10]. We targeted the patient management for better glycemic control and referred her to physiotherapy for management. Intensive glycemic control, physiotherapy for passive palmar stretching, and occupational therapy have shown to improve the range of movement and hand functionality [11].

Generally, since LJM is an association or complication of chronic uncontrolled diabetes, the best preventive measure is to target well-controlled glycemic range and to involve the family to prepare the appropriate environment for this target starting from the early diagnosis stage.

Over the last two decades, many experimental studies on medications with AGE inhibitory properties targeting AGE cross-links have shown beneficial effects. However, human trials still need to prove the safety of these medications which are not in clinical use yet [12–14].

## 4. Conclusion

We presented a child with bilateral hand limited joint mobility limitation as one of the rare type 1 DM complications as an important indicator of the microvascular complications in this age group, which mandates to be examined for during the outpatient visits of diabetic children for early detection and management. Good glycemic control with clear HbA1c and daily glucose range targets are still the mainstay of prevention of such complication and its associations. The available therapeutic options include symptomatic relief and hand's function preservation through physiotherapy awaiting solid and extensive clinical trials to prove the safety and usefulness of the new AGE inhibitors.

## Data Availability

The data used to support the findings of this study are available from the corresponding author upon request.
